# Lichen planopilaris in 24 African American women

**DOI:** 10.1097/JW9.0000000000000141

**Published:** 2024-04-02

**Authors:** Allen S. W. Oak, Kevin Yang, Sivani B. Reddy, Carly A. Elston, Tiffany T. Mayo

**Affiliations:** a Department of Dermatology, University of Pennsylvania, Philadelphia, Pennsylvania; b Department of Dermatology, University of Alabama at Birmingham, Birmingham, Alabama

**Keywords:** African American, alopecia, lichen planopilaris, scaring alopecia, trichoscopy

**What is known about this subject in regard to women and their families?**

- Lichen planopilaris (LPP) mainly affects women, and hair loss can be a major psychosocial stressor that negatively affects patients and their families.
- Until now, scarring alopecia in Black women has been assumed to be most likely central cicatricial centrifugal alopecia.

**What is new from this article as messages for women and their families?**

- This article introduces LPP as a diagnosis that should also be considered in this patient population and also describes classic findings of LPP and a distinct pattern that we refer with the term “arrowhead distribution,” which may be indicative of a progressive scarring alopecia.
- Arrowhead sign may be a useful clinical clue to the diagnosis of progressive LPP in Black patients.
- When LPP is suspected, biopsy can confirm and avoid treatment delays and ultimately improve patient outcomes.

## Introduction

Lichen planopilaris (LPP) is a primary cicatricial alopecia. Classically, LPP presents with multifocal hair loss, especially at the vertex and parietal scalp, with trichoscopic signs of perifollicular erythema and scale.^1,2^ Frontal fibrosing alopecia (FFA) is a variant of LPP involving the frontal hairline. Less common patterns of LPP recently recognized include fibrosing alopecia in a pattern distribution with shared features of androgenetic alopecia and a diffuse pattern of LPP with involvement throughout the scalp.^3,4^ LPP is mostly reported in postmenopausal White women; however, limited data exist on LPP in Black patients. Here we report clinical characteristics of biopsy-proven LPP in 24 African American females in a single center.

## Methods

An institutional review board–approved retrospective chart review of African American adults with clinical and histologic diagnosis of LPP (Supplementary Figure 1, http://links.lww.com/IJWD/A44) was performed between 2017 and 2021.

## Results

Twenty-fourAfrican American females were included (Table 1). Age ranged from 26 to 73 years old with an average of 48.4 ± 12.9. All patients had clinical signs and/or symptoms consistent with LPP. No evidence of additional cutaneous or nail involvement was observed. Hepatitis C virus labs were not available for all patients and were excluded from the analysis. Corresponding loss of eyebrows and eyelashes was seen in 3 patients. Histologic characteristics of androgenetic alopecia were seen in addition to LPP in 12 patients (50%), in line with the known prevalence of androgenetic alopecia in the general population. The majority (22/24, 91.7%) of patients had vertex involvement, and 17 (70.8%) had both frontal and vertex involvement. Three patients had complete involvement of the frontal and vertex scalp with relative sparing of the occipital scalp. The corresponding trichoscopy demonstrated classic findings of LPP including loss of ostia (8/24, 33.3%), perifollicular erythema (16/24, 66.7%), and perifollicular scale (15/24, 62.5%). All 24 patients lacked clinical pustules suggestive of folliculitis decalvans. The most common comorbid condition was hypertension (10/24, 41.7%).

**Table 1:** Baseline characteristics of each patient

Patient no.	Age (yr)	Distribution of scalp involvement	Trichoscopy	Loss of eyebrows and/or eyelashes?	Comorbid conditions	Biopsy results	Clinical differential	Arrowhead sign?
1	73	Frontal, temporal, parietal	Perifollicular erythema, loss of follicular ostia	No	HTN	LPP	FFA vs traction alopecia	N/A^a^
2	41	Temporal, vertex	Perifollicular erythema and scale, areas of decreased follicle density	No	None	LPP/AGA overlap	LPP > CCCA	Yes
3	68	Frontal, temporal, vertex	Perifollicular erythema	No	HTN, hyperlipidemia, obstructive sleep apnea	LPP	LPP > CCCA	No
4	64	Frontal, parietal, vertex	Perifollicular scale	No	GERD, atrial fibrillation	LPP	LPP	No
5	61	Frontal, vertex	Perifollicular erythema, loss of follicular ostia	No	HTN, meningioma	LPP	CCCA vs LPP	Yes
6	58	Frontal, temporal, parietal, vertex (diffuse and confluent)	Loss of follicular ostia	No	HTN, GERD	LPP	Scarring alopecia vs alopecia totalis	N/A^a^
7	38	Frontal, temporal, parietal, vertex	Perifollicular erythema and scale, loss of follicular ostia	No	None	LPP	CCCA vs LPP	Yes
8	44	Frontal, temporal, parietal, vertex	No perifollicular erythema, dots, or scaling	No	Morbid obesity, unprovoked deep vein thrombosis	LPP/AGA overlap	AA vs LPP vs CCCA	No
9	31	Frontal, vertex (diffuse and confluent)	Perifollicular erythema and scale, peripilar white halos, honeycomb pigmented matrix	No	Hypercholesterolemia, polycystic ovary syndrome	LPP/AGA overlap	AA vs LPP vs CCCA	N/A^a^
10	33	Temporal, parietal, vertex	Background erythema and perifollicular scale	No	GERD, migraine	LPP	CCCA vs LPP	Yes
11	50	Frontal, vertex	Perifollicular erythema and scale, loss of follicular ostia	No	HTN, hyperlipidemia, obesity, GERD	LPP	CCCA vs LPP	Yes
12	54	Frontal, temporal, parietal, vertex (diffuse and confluent)	Perifollicular erythema, loss of follicular ostia	No	SLE, diverticulitis s/p partial colon resection	LPP	CCCA vs LPP	N/A^a^
13	60	Frontal, vertex	Loss of follicular ostia, perifollicular scale	No	HIV (on emtricitabine/rilpivirine/tenofovir)	LPP/AGA overlap	AGA vs LPP	Yes
14	39	Frontal, vertex	Perifollicular erythema and scale	No	None	LPP	LPP vs CCCA vs AGA	Yes
15	54	Parietal, vertex	Perifollicular erythema and scale	No	None	LPP/AGA overlap	CCCA vs LPP	Yes
16	36	Frontal, parietal, vertex	Perifollicular erythema and scale, patchy areas of follicular dropout	Loss of eyebrows and eyelashes	None	LPP/AGA overlap	LPP vs CCCA vs AGA	NP
17	60	Frontal, vertex	Loss of follicular ostia	Loss of eyebrows	HTN	LPP/AGA overlap	LPP vs CCCA vs AGA	NP
18	53	Frontal	Perifollicular erythema and scale	No	HTN, hypercholesterolemia, cutaneous sarcoidosis	LPP	LPP vs traction alopecia	N/A^a^
19	51	Frontal, temporal, vertex	Perifollicular erythema	No	HTN, sarcoidosis, asthma	LPP/AGA overlap	CCCA vs LPP	Yes
20	32	Frontal, temporal, parietal, vertex	Perifollicular erythema and scale	No	HTN	LPP	LPP	Yes
21	35	Parietal, vertex	Perifollicular erythema and scale, perifollicular blue-gray halo	No	Tachycardia	LPP/AGA overlap	LPP vs CCCA vs AGA	Yes
22	44	Vertex	Perifollicular scale	No	None	LPP/AGA overlap	CCCA vs LPP	NP
23	56	Frontal, vertex	No perifollicular erythema, dots, or scaling, hair miniaturization	No	HTN	LPP/AGA overlap	LPP/FFA vs AGA vs CCCA	Yes
24	26	Frontal, temporal, vertex	Perifollicular erythema and scale	Loss of eyebrows	None	LPP/AGA overlap	CCCA vs LPP	No

AA, alopecia areata; AGA, androgenetic alopecia; CCCA, central cicatricial centrifugal alopecia; FFA, frontal fibrosing alopecia; GERD, gastric esophageal reflux disease; HIV, human immunodeficiency virus; HTN, hypertension; LPP, lichen planopilaris; NP, no photographs available; SLE, systemic lupus erythematosus; s/p, status post.

a Unable to evaluate the patient for an arrowhead sign due to diffuse and confluent hair loss of vertex and frontal scalp.

**Fig. 1.**
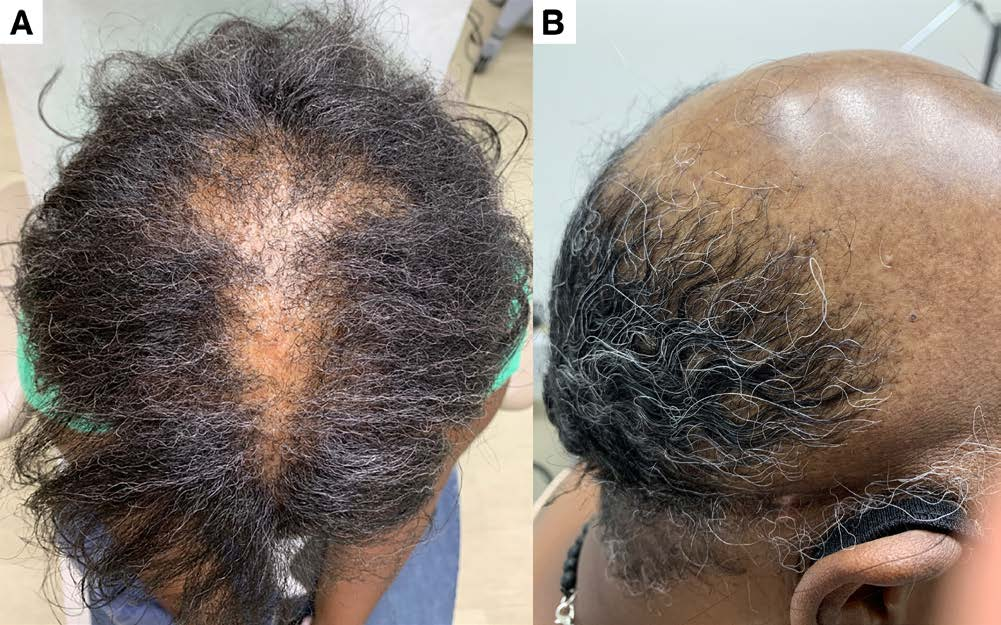
(A) Lichen planopilaris (LPP) involvement in an “arrowhead” distribution. Patch of scarring alopecia consistent with LPP distributed over the frontal and vertex scalp with “arrowhead” distribution in an African American female. (B) LPP with complete involvement of the frontal and vertex scalp. Complete scarring alopecia involving the frontal and vertex scalp with relative sparing of the occipital scalp.

It was noted that many patients with vertex involvement had a characteristic triangular shape distribution rather than a round centrifugal distribution (Fig. 1A). Patients with clinical photographs were evaluated for this finding. Patients with diffuse and confluent loss of the vertex and frontal scalp more characteristic of FFA were excluded. Twelve of the remaining 16 patients demonstrated this characteristic distribution of alopecia with morphology similar to that of an “arrowhead.”

## Discussion

Classic LPP in Caucasian patients has been described to involve the vertex and parietal scalp, and a large cohort of LPP patients from Iran also most commonly involved the parietal scalp.^1^ The pattern of frontal and vertex scalp involvement in our patients was striking and distinct from FFA, which typically involves the frontotemporal scalp, and fibrosing alopecia in a pattern distribution, which presents in a classic androgen-dependent or “Christmas tree” distribution.

Patients were noted to have alopecia ranging from mild to complete alopecia of the frontal and vertex scalp (Fig. 1B). A distinct pattern, which we refer with the term “arrowhead” distribution, was seen in many patients where scarring occurred in a triangular distribution from the vertex toward the frontal scalp. This pattern differs from the typical round centrifugal distribution classically associated with central centrifugal cicatricial alopecia. “Arrowhead” pattern may not be pathognomonic for LPP, as many Black patients with LPP do not demonstrate this pattern; however, it may be indicative of progressive scarring alopecia. More research is needed to understand patterns of scarring alopecia.

The differential diagnosis of vertex cicatricial alopecia in Black patients is commonly limited to central centrifugal cicatricial alopecia, though the full spectrum of scarring alopecia is seen in this patient population. Based on our findings, LPP should be strongly considered if classic dermatoscopic findings are noted. The pattern may differ from classic LPP. Predominant involvement of both vertex and frontal scalp was seen in this cohort of African American women with LPP. Arrowhead sign may be a useful clinical clue to the diagnosis of progressive LPP in Black patients.

When LPP is suspected, a biopsy can confirm and avoid treatment delays. Accurate diagnosis may improve awareness and outcomes for scarring alopecia. More research is needed to better understand and distinguish alopecias in Black patients.

## Conflicts of interest

The authors made the following disclosures: T.T.M. has served as an investigator or consultant for Abbvie, Arcutis, Acelyrin, BMS, ChemoCentryx, Eli Lilly, Galderma, Janssen, Leo Pharma, Novartis, Pfizer, and Procter and Gamble, UCB. A.S.W.O., K.Y., S.B.R., and C.A.E. declare no conflicts of interest.

## Funding

None.

## Study approval

The authors confirm that any aspect of the work covered in this manuscript that has involved human patients has been conducted with the ethical approval of all relevant bodies. The study protocol was reviewed and approved by the University of Alabama at Birmingham Institutional Review Board (approval no. 300005995).

## Author contributions

TTM and CAE: Participated in research design, performance of the research, the writing of the article, and data analysis. ASWO and KY: Participated in the performance of the research, the writing of the article, and data analysis. SBR: Participated in the performance of the research. All authors have seen and approved the final version.

## Patient consent

Informed, written consent was received from all patients for whom photographs are present in the manuscript.

## Data availability

Data that support the findings of this study are available from the corresponding author (TTM) upon reasonable request.

## Supplementary data

Supplementary material associated with this article can be found at http://links.lww.com/IJWD/A44.

## Supplementary Material


