# Supplementary material for: Lichen planopilaris in 24 African American women
Source: Int J Womens Dermatol. 2024 Apr 2;10(2):e141. doi: 10.1097/JW9.0000000000000141 (PMC10986913; doi:10.1097/JW9.0000000000000141)
Supplement: Supplementary file 1 [file jw9-10-e141-s001.pdf]

Supplement

eFigure 1.

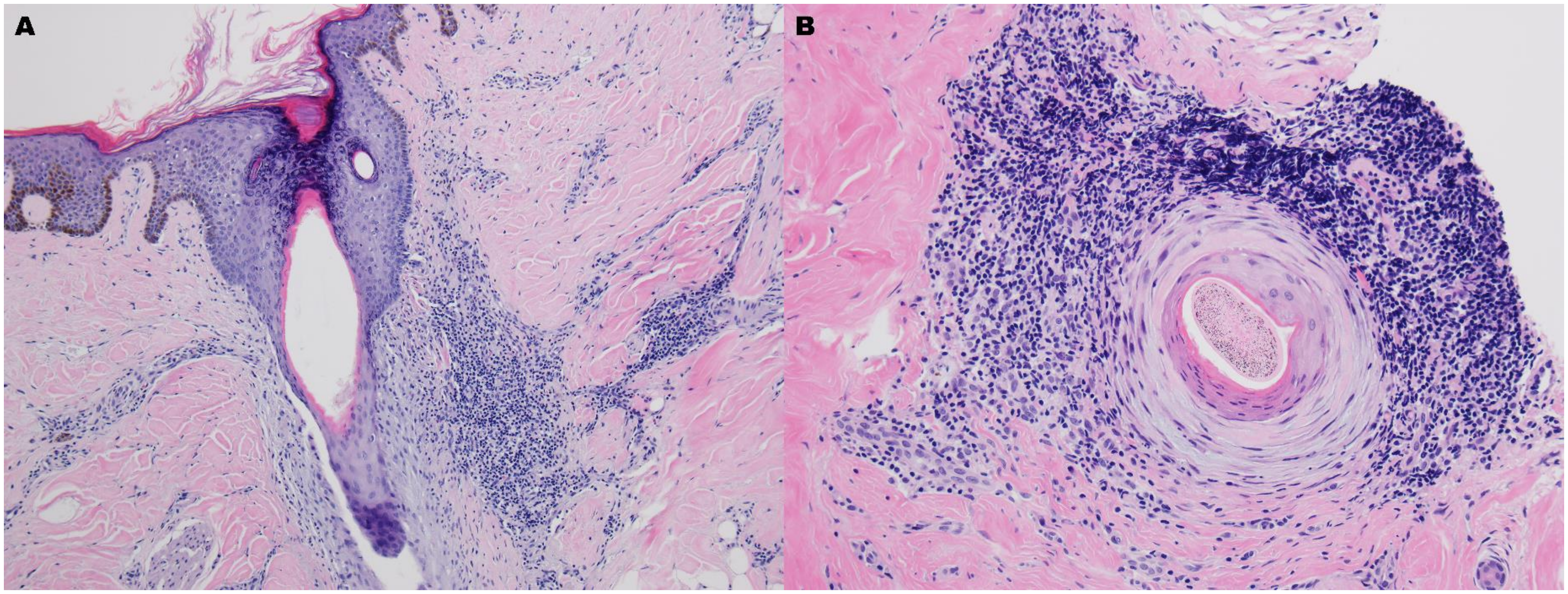

eFigure 1. Lichen planopilaris histological images.

(A) Vertical and (B) horizontal sections of representative punch biopsies show a decreased number of hair follicles with wedge-shaped scarring, loss of sebaceous glands and a brisk lymphocytic infiltrate surrounding the follicular infundibulum with associated dyskeratosis and mucinous fibroplasia. Original magnifications: (A) 10x; (B) 20x.
